# Exposure of Soil Microbial Communities to Chromium and Arsenic Alters Their Diversity and Structure

**DOI:** 10.1371/journal.pone.0040059

**Published:** 2012-06-29

**Authors:** Cody S. Sheik, Tyler W. Mitchell, Fariha Z. Rizvi, Yasir Rehman, Muhammad Faisal, Shahida Hasnain, Michael J. McInerney, Lee R. Krumholz

**Affiliations:** 1 Department of Botany and Microbiology, University of Oklahoma, Norman, Oklahoma, United States of America; 2 Institute for Energy and the Environment, University of Oklahoma, Norman, Oklahoma, United States of America; 3 Department of Microbiology and Molecular Genetics, University of the Punjab, Lahore, Pakistan; Argonne National Laboratory, United States of America

## Abstract

Extensive use of chromium (Cr) and arsenic (As) based preservatives from the leather tanning industry in Pakistan has had a deleterious effect on the soils surrounding production facilities. Bacteria have been shown to be an active component in the geochemical cycling of both Cr and As, but it is unknown how these compounds affect microbial community composition or the prevalence and form of metal resistance. Therefore, we sought to understand the effects that long-term exposure to As and Cr had on the diversity and structure of soil microbial communities. Soils from three spatially isolated tanning facilities in the Punjab province of Pakistan were analyzed. The structure, diversity and abundance of microbial 16S rRNA genes were highly influenced by the concentration and presence of hexavalent chromium (Cr (VI)) and arsenic. When compared to control soils, contaminated soils were dominated by *Proteobacteria* while *Actinobacteria* and *Acidobacteria* (which are generally abundant in pristine soils) were minor components of the bacterial community. Shifts in community composition were significant and revealed that Cr (VI)-containing soils were more similar to each other than to As contaminated soils lacking Cr (VI). Diversity of the arsenic resistance genes, *ars*B and *ACR3* were also determined. Results showed that *ACR3* becomes less diverse as arsenic concentrations increase with a single OTU dominating at the highest concentration. Chronic exposure to either Cr or As not only alters the composition of the soil bacterial community in general, but affects the arsenic resistant individuals in different ways.

## Introduction

Anthropogenic metal contamination is a problem frequently encountered near long-term industrialized areas [1]. In Pakistan, the leather industry has left soils surrounding production facilities contaminated with chromium (VI & III) and arsenic. Accumulation of Cr (VI) and arsenic in soils is of concern due to their mutagenic and carcinogenic properties in humans [2], [3]. Geochemical cycling of chromium and arsenic can be microbially mediated in both aerobic [4], [5] and anaerobic [6], [7] systems. Within the environment, chromium is found as chromite [Cr(III)] or chromate [Cr(VI)] [8]. Chromite is less toxic and poorly mobile due to its low water solubility at neutral pH and its sorption characteristics [8]. In contrast, chromate is acutely toxic, mutagenic, teratogenic and carcinogenic (USEPA, 1998b). It is soluble in water and mobile in soils and sediments [8]. Cr (VI) resistance is thought to involve the use of a chromium efflux pump [9], [10] while detoxification (*i.e.* reduction to Cr (III)) is less understood but several genes have been proposed [11].

Inorganic arsenic, much like chromium, is primarily found in two valence states in the environment, pentavalent [arsenate, As (V)] and trivalent [arsenite- As (III)] [12]. In contrast to chromium, the reduction of arsenate to arsenite increases the solubility and toxicity, as arsenite is uncharged at pH <9 [13]. Several resistance/reduction mechanisms for arsenate are well described (for a review, see Oremland and Stolz 2003; 2005). The *ars* genes have been shown to confer arsenate resistance via enzymatic reduction followed by efflux of arsenite through a transmembrane pump [14]–[16]. The *ars* operon has been found primarily in bacteria [15], [17]. A functionally similar group of genes (*ACR*) were originally observed in fungi [18], [19] until recent discovery of an efflux pump-encoding gene genetically similar to *ACR* in several bacterial species [14], [20]. To date, many of the pure-culture isolates resistant to or capable of transforming chromium or arsenic are predominantly associated with the bacterial phyla *Proteobacteria*, *Firmicutes*, and *Actinobacteria* [8]. However, due to the dearth of bacterial isolates with known As & Cr resistance mechanisms, it is unclear whether resistance to and transformation of chromium and arsenic is common in underrepresented phyla or underrepresented clades within well-characterized phyla.

The diversity of soil microbial communities is exceedingly rich [21]–[25]; however it is still not possible to predict how microbial communities will respond when exposed to metals, as in some cases diversity (*i.e.* the richness and evenness of species within a sample) and microbial biomass have been observed to decrease [1], [26] while in others, they show no correlation [27]. In systems acutely exposed to contamination, diversity may be maintained within the community through either natural resistance [28] or the ability of organisms to lay dormant until favorable growth conditions return [29]. Conversely, chronic exposure to contamination will likely have deleterious effects on the structure and ultimately the function of the community, as dormancy may not be a useful survival option. The degree of species loss will likely be a function of the mobility of resistance genes (*i.e.* horizontal gene transfer) [30] and the behavior of the metal species in the environment [31]. Soil microbial communities are known to share fewer species as distance between sites increases [24], [32] However, it has been observed that landscape and pH can select for similar communities despite spatial separation [33]. In the face of long-term exposure to metals, restructuring of microbial communities is likely [34] but it is unclear which groups of microorganisms are more tolerant to chromium and arsenic contamination and how metal resistance genes respond. Therefore we sought to understand the effects of chronic chromium and arsenic contamination on soil bacterial communities from three spatially distant, long-term leather production areas in the Punjab province of Pakistan. We found that in the presence of metals novel *Proteobacteria* were abundant community members and that arsenic concentration dramatically influenced the diversity of arsenic resistance genes.

## Methods

### Sampling Site, Collection and DNA Extraction

Three sampling sites near the University of Punjab in Lahore, Pakistan were selected based on their longstanding history (>40 years) of industrial waste disposal primarily from the chrome tanning industry. No specific permits were required for the sampling of these selected sample sites, and they were not privately owned or protected in any way. The sampling and studies performed also did not affect or involve any endangered or protected species in the area. The three sites selected were spatially separated by 50–200 km and located within or near the cities of Lahore (Control: 31 44′44.24",74 15′54.61"; Contaminated: 31 44′44.32",74 15′52.92"), Sialkot (Control: 32 28′21.10", 74 30′57.53"; Contaminated: 32 28′18.57", 74 30′59.57"), and Kasur (Control: 31 06′03.89", 74 27′42.45"; Contaminated: 31 06′18.53", 74 27′34.17") (see Table 1 for habitat type). We employed a paired sampling design, such that control soil samples were collected near contaminated sites (<0.5 km) and had similar soil properties. Contamination and soil type were taken into consideration and minimized as much as possible when choosing control and contaminated site pairs. Soil was collected from each site in 0.1 m^2^ blocks using a sterile shovel, placed into gamma-sterilized polyethylene bags, transported on ice back to the laboratory, and stored at −20^o^C. Soil blocks were broken apart, homogenized and partitioned for DNA extraction and soil characterization. Soil texture, chemistry, and metal analyses were performed by the Pakistan Council Scientific and Industrial Research (PCSIR) (Lahore, Pakistan). Metals were extracted using the microwave-assisted ISO 11466:1995 standard method. Soil (10 g) was dispersed into 20 ml *aqua regia* containing a 3∶1 ratio of concentrated HNO_3_:HCl v/v. Soils were incubated for two hours followed by microwave digestion for 15 min. Total extractable chromium and arsenic were analyzed using inductively coupled plasma atomic emission spectroscopy (ICP-AES). Chromium VI was extracted from soil with distilled water and assayed spectrophotometrically at 540 nm using the diphenylcarbazide method [35]. Soil pH was measured by creating a soil slurry of distilled water and 10g soil, followed by room temperature incubation (1 hr) with intermittent shaking. Soil texture was determined by particle size distribution ISO 11277:2009. DNA was extracted from 7.0 g of homogenized soil taken from the upper 20 cm of the block from each site using a MoBio PowerMax soil DNA isolation kit (MoBio, Carlsbad, CA, 92010, USA). DNA was precipitated and dried according to the manufacturer’s directions and then shipped to the University of Oklahoma for further analysis. All DNA was resuspended in 2.0 ml of sterile nuclease free water.

### Pyrosequencing and Quantitative PCR

PCR libraries were generated using the modified 338 (5′ACHCCTACGGGWGGCWGC) forward and 518 (5′ACCGMSGKKGCTGGCAC) reverse primers [25]. Modifications to the primer set were done according to Hamady *et al.* [36], which included: adding the B-adapter and a unique 8 base barcode to the 5′ end of the 518 reverse primer while the A-adapter was added to the 5′ end of the forward primer (sequences for A and B FLX adapters were taken from http://www.454.com, 454 Life Sciences, Branford, CT). Pseudo replicates from each site were created for DNA from control and contaminated sites by using two separate barcodes per site. DNA from each site was PCR amplified in four 100-µl reactions that contained (final concentration): 4 µl of 1/10 diluted DNA, 1x Hot Start buffer (Fermentas, Glen Burnie, MA, USA), 2.0 mM MgCl_2_, 0.2 mM deoxynucleoside triphosphate mixture (Fermentas), 3.0 U Maxima Hot Start *Taq* DNA polymerase (Fermentas), and 500 nM of the forward and reverse primer. The PCR amplification protocol was as follows: 5 min at 95°C, followed by 30 cycles of 95°C for 1 min, 54°C for 1 min, and 72°C for 1 min with a final extension of 15 min at 72^o^C. PCR was performed using an Applied Biosystems GeneAmp 9700 (Applied Biosystems, Carlsbad, CA, 92008, USA) PCR reactions were screened using gel electrophoresis and positive reactions were pooled (site and treatment kept separate) and concentrated using standard sodium acetate/ethanol method. Two volumes of 100% ethanol and 1/10 volume of 3M sodium acetate (pH = 4.5) were added to pooled PCR products, incubated at −80^o^C for 30 min, and centrifuged for 30 min at 4^o^C. PCR precipitate was dried and resuspended in 50 µL of sterile nuclease free water. Concentrated PCR products were screened using a 2% agarose gel and bands of appropriate size were extracted and recovered using a Freeze ‘N Squeeze DNA gel extraction spin column (Bio-Rad, Hercules, CA, 94547, USA). Purified products were quantified using a NanoDrop 1000 (NanoDrop products, Wilmington, DE, 19810, USA) and pooled at equal concentrations. PCR products were then sent to Engencore (http://engencore.sc.edu/) for pyrosequencing using FLX chemistry.

A full description of Quantitative PCR (QPCR) methods for the universal bacterial primer set 338F–518R can be found in Sheik *et al.* [25]. Briefly, a plasmid standard containing the 338–518 16S rRNA gene fragment from *Escherichia coli* was quantified using a NanoDrop 1000 (NanoDrop Products), diluted to 10^9^ copies per µl and then ten-fold serially diluted. The serial dilutions (10^4^–10^9^) were used to generate a standard curve to calculate the gene copy number in the environmental samples. QPCR was performed in triplicate on each soil using the Bio-Rad MyIQ real-time PCR system (Bio-Rad, Hercules, CA). Each reaction mixture (25-µl total volume) consisted of 12.5 µl IQ SYBR Green Supermix (Bio-Rad), 9.5 µl of water, 1.0 µl (10 µM) of each primer, and 2.0 µl of diluted DNA. QPCR generally followed a standard two-step protocol consisting of 5 min at 95°C, followed by 40 cycles of 95°C for 30 s, 56°C for 30 s, and 72°C for 30 s. Thermocycler protocols were optimized to achieve a qPCR amplification efficiencies of nearly −3.3.

### Targeted PCR of ACR3 and arsB Genes from Soil Community DNA

Amplification of the gene encoding the *ACR*3 As (III) efflux pump was performed using two separate degenerate primer sets [14]. The primer set acr5F (5′-TGATCTGGGTCATGATCTTCCCVATGMTGVT-3′) and acr4R (5′-CGGCCACGGCCAGYTCRAARAARTT-3′) was used to amplify the *ACR3* gene from the subclade two-arsenite efflux pump. Primers darsb1F (5′-GGTGTGGAACATCGTCTGGAAYGCNAC-3′) and darsb1R (5′-CAGGCCGTACACCACCAGRTACATNCC-3′) [14] were used on soil DNA to amplify the *ars*B genes from the community and isolate DNA. All described PCR reactions contained (final concentration): 0.2 µM of each primer, 1.5 U of Dreamtaq DNA polymerase (Fermentas, Glen Burnie, MA, USA), 1x Greenbuffer PCR buffer, 10 mM DNTPs and 2 µL of extracted soil DNA diluted 1/10 with nuclease free H_2_O to alleviate inhibition. This mixture was then amplified with a 5 min denaturation at 94°C followed by 35 cycles of 94°C for 45 seconds, annealing at 57–52°C with a 0.5°C decrement per cycle during the first 10 cycles (45 seconds) and then 72°C for 30 seconds with a final extension of 72°C for 7 minutes [14]. All PCR products were purified using a PCR purification kit (Invitrogen, Carlsbad, CA, USA) Target specificity of PCR primer pairs was done before further analysis.

### Clone Libraries, Sequencing and Phylogenetic Analysis

Clone libraries were generated from the PCR amplicons of the *ACR3* and *arsB* genes at each site. Clones were generated using a TOPO-TA clone kit (Invitrogen, Carlsbad, CA, USA) utilizing the optional blue/white screening test on LB media supplemented with ampicillin and S-Gal. Clones were randomly selected and checked for inserts by PCR prior to sequencing. Clone libraries, consisting of 48 clones for each gene and site, were replicated from stocks before being sequenced by Microgen (Microgen, Oklahoma City, OK). Sequencing was performed using an ABI 3730xl capillary sequencer (Applied Biosystems) Sequences underwent quality screening, primer removal, and binning into Shared Operational Taxonomic Units (OTUs) at a cutoff level of 90% with DNAstar (DNAStar inc., Madison, WI, USA). Quality OTUs were taxonomically identified using BLASTx (National Center for Biotechnology Information, Bethesda, MD, USA), and these results were combined with sequences identified in Cai *et al*. [30] for phylogenetic comparison. Sequences with poor sequence alignment, no BLAST hits, or BLAST hits to genes that did not encode *ACR3* or *arsB* were removed from downstream analyses. OTUs and known sequences were aligned using Muscle [37] and then imported into MEGA 5 [38] for construction of phylogenetic dendrograms using the Neighbor-Joining algorithm with the following parameters: Jukes-Cantor correction model for nucleotides and 1000 Bootstraps.

### Analysis of ACR3 and *arsB* Genes from Isolated Organisms

Several arsenic tolerant bacterial isolates were cultured from arsenic contaminated sites in the same region as the original soil samples. DNA was extracted from these isolates and screened for the *ACR*3(2) as well as the *arsB* genes utilizing the PCR methods described above. Positive PCR reactions were sequenced. The sequences were then used in the phylogenetic analysis of the clone library for comparison to the data obtained from the community DNA.

### T-RFLP Analysis of Diversity

DNA from each contaminated site was PCR amplified using methods outlined above with the darsB1R (5′) primer was labeled with 6-carboxy-fluorescine (FAM). PCR products were screened by gel electrophoresis and subsequently purified using a PCR purification kit (Invitrogen, Carlsbad, CA, USA). Purified PCR products were then digested with HaeIII (New England BioLabs, Ipswich, MA) at 37°C for 3 hours, and then the enzyme was inactivated at a temperature of 65°C for 15 minutes. The restriction digests were quality checked by gel electrophoresis before being sent for terminal fragment analysis (Microgen, Oklahoma City, OK). The internal size standards LIZ 600 (Applied Biosystems) were added by the sequencing facility prior to peak quantification. T-RFLP data were processed using the Peak Scanner software (Applied Biosystems, Carlsbad, CA, USA) to determine peak height and area. The diversity of T-RFLP patterns was estimated by calculating Shannon indices (Shannon Calculator, Chang Bioscience, Castro Valley, CA).

### Data Processing of Pyrosequencing Reads

Raw pyrosequencing reads were binned by barcode, quality screened by using an average minimum quality score of twenty, and trimmed of primer sequence using the RDP pyrosequencing pipeline (http://pyro.cme.msu.edu/index.jsp). Sequence reads were then imported into the phylogenetic software package Mothur version 1.14.0. 64 bit [39] (http://www.mothur.org) for OTU (Operational Taxonomic Unit) generation, diversity estimates and classification. Full descriptions of workflows can be found within the Mothur manual. Before alignment, sequences with more than eight homopolymer nucleotides and outside of our length requirement (140–180 bases) were removed. Within Mothur, sequences were aligned to the Silva core sequence set using the NAST algorithm [40]. Sequences were then chimera checked with Chimera Slayer, sequence distances were calculated with no penalization for end gaps, OTUs were clustered using the furthest neighbor algorithm and were then classified within Mothur using Greengenes taxonomy and the Silva database. OTUs_0.03_ (Species level Operational Taxonomic Units) taxonomy was obtained by consensus using a cutoff of 60%. Alpha diversity metrics (Abundance-based coverage estimator (ACE) [41], Chao (estimates total species richness) [42], Shannon Index (calculates diversity within a sample) [43], rarefaction curves and rarified phylogenetic diversity) were also calculated with Mothur. In order to calculate beta diversity with Unifrac [44] and Faith’s phylogenetic diversity [45], [46], a phylogenetic tree was generated with the program FastTree version 2.1 [47]. To build the phylogenetic tree OTUs_0.03_ from each sample were aligned to the Silva database and lane masked. Unifrac analysis was performed using the Fast Unifrac web interface [48]. Pearson correlation coefficients for environmental factors and diversity estimates were calculated in excel with the CORREL correlation function. Significance of the correlation was tested against a null Student’s *t*-test model using a two-tailed distribution to assess significance (P≤0.05) [49]. Redundancy analysis (RDA) was performed in R (http://cran.r-project.org/) using the vegan package [50] with normalized OTU abundance and environmental chemical data.

Pyrosequencing data has been deposited at NCBI Sequence Read Archive accession number SRA026044. Clone library sequences from *ACR*3 and *ars*B have been deposited in GenBank under the following accession numbers *ACR*3 (JQ409060-JQ409108) and *ars*B (JQ608478–JQ608486).

## Results

### Geochemistry of Sampling Sites

**Figure 1:**
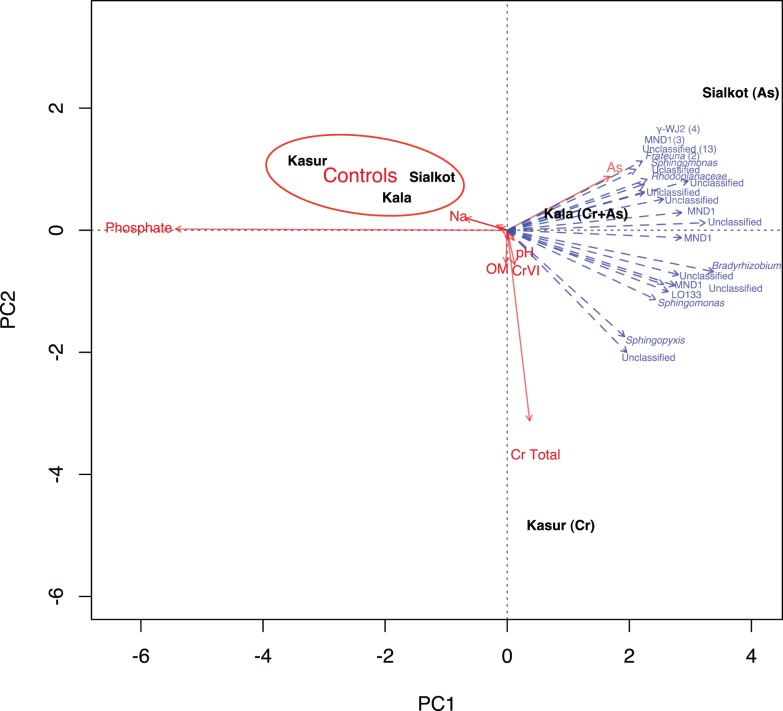
Redundancy analysis (RDA) of soil geochemical data and identification of significant *Proteobacterial* clades. Geochemical data from Table S1 were used to generate PCA and OTU abundance was used to generate the RDA. Factors and bacterial groups that had little influence on any of the sites (*i.e.* clustered near the center axis) or were similarly clustered were removed for clarity.

Contaminated soils differed in the presence and amounts of Cr (VI) and As (Table 1). Chromium was detected in all of the contaminated and control sites, the majority of which was Cr (III) (Table 1). Cr (VI) and As were not detected in any of the control soils. Despite the extensive use of both arsenic and chromium by the tanning industry, the combination of arsenic and chromium were each only detected in two of the three contaminated soils. The Kasur soil had the highest levels of Cr (VI) and total chromium, but undetectable levels of arsenic. The Sialkot soil on the other hand, contained the highest level of arsenic, but no detectable Cr (VI) and little total chromium. The Kala Shah Kaku soil contained high levels of Cr (VI), total chromium and arsenic. The Kala Shah Kaku and Kasur contaminated soils also had the highest pH, clay content, and total chromium levels (Table S1). Despite being spatially separated by 50–200 km, soil textures were similar with the exception of the contaminated Kala Shah Kaku and Kasur soils, which contained a higher silt and clay content than the other contaminated and uncontaminated soils (Table S1). Principal Components Analysis revealed that much of the variance in soil properties and chemistry between the contaminated sites was associated with pH, arsenic, total chromium, and Cr (VI) levels, while control soils were correlated with di and monovalent cations, bicarbonate, and phosphate concentrations (Figure 1). The pH of contaminated sites was slightly more alkaline (pH 7.0–8.0) than the paired control sites (6.4–6.8).

**Table 1:** Location and key geochemical data of contaminated and control study sites.

Site	Contamination Status	pH	As (mg kg^−1^)	Cr (VI) (mg kg^−1^)	Total Cr (g kg^−1^)
Kala Shah Kaku	Control	6.5	BD^1^	BD	3.82
	Cr (VI) and As contaminated	7.4	6.6	1.2	6.24
Kasur	Control	6.4	BD	BD	1.61
	Cr (VI) contaminated	8	BD	4.21	24.78
Sialkot	Control	6.8	BD	BD	2.36
	As contaminated	7	13.9	BD	0.23

Additional soil and chemical analyses are presented in Table S1.

1 BD: Below detection. Detection limit for chromium is 0.004 mg kg^−1^ and 0.008 mg kg^−1^ for arsenic.

### Microbial Community Response to As and Cr Contamination Revealed by Pyrosequencing

**Figure 2:**
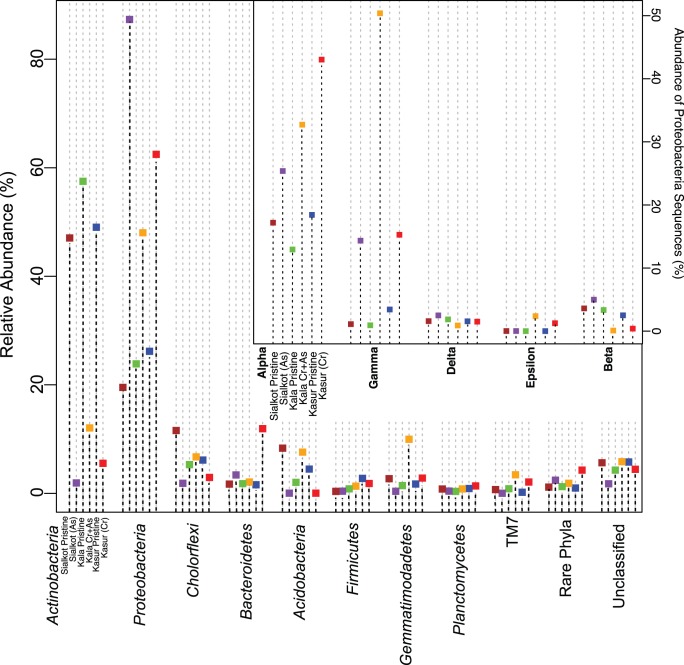
Phylogenetic distribution of the dominant phyla identified by pyrosequencing. The dominant phyla identified in pyrosequencing libraries are ordered by relative dominance in the pyrosequencing libraries. Each sampling site is represented by color and order is consistent for all phyla. Distribution of proteobacterial classes is described in the inset panel. Abundance was based on the total number of proteobacterial sequences recovered at each site. Little variation between sub-samples for each site was observed thus error bars were left out of figure for clarity.

From the three-paired sampling sites, a total of 232,216 sequences were obtained after quality screening, resulting in an average of 15,000–28,000 sequence reads per sampling site (Table 2). One subsample from Kala Shah Kaku (1a) failed to generate enough sequence tags (approximately 1000) after the quality screening and was not further analyzed. Comparative analysis of the control and contaminated sites revealed several large shifts in the relative abundance of many of the dominant phyla. Uncontaminated sites all shared similar phylum level profiles, whereby *Actinobacteria*, *Proteobacteria*, and *Chloroflexi* were the most abundant phyla present (Figure 2). This pattern is similar to phylum level profiles generated by other pyrosequencing studies of soil [23], [25], [51], suggesting that the chosen control sites provided an adequate baseline for comparison to contaminated soils. The contaminated soils all had a similar phylum-level abundance profile that was drastically different from their paired controls. In contrast to the control soils where *Actinobacteria* was the dominant phylum, *Proteobacteria* was the dominant phylum in contaminated soils (Figure 2). Within the *Proteobacteria, Alphaproteobacteria* or *Gammaproteobacteria* were the most abundant classes in all soils. In soils containing either As (Sialkot) or Cr (VI) (Kasur) only, *Alphaproteobacteria* was the most abundant class. However, when both As and Cr (VI) (Kala Shah Kaku) were present, *Gammaproteobacteria* was the most abundant class (Figure 2). *Betaproteobacteria* and *Deltaproteobacteria* and *Epsilonproteobacteria* were detected at all sites, but less abundant than *Alphaproteobacteria* or *Gammaproteobacteria* (Figure 2). *Chloroflexi* were more abundant than *Acidobacteria* in control soils (Figure 2). In contaminated soils with Cr (VI) (Kala Shah Kaku and Kasur), *Acidobacteria* were rare members of the community constituting less than 1% of their respective libraries. In Sialkot soils contaminated with arsenic but not Cr (VI), *Acidobacteria* was more abundant than its paired control. Several novel proteobacterial groups MND1, LO133, and WJ2 were identified by redundancy analysis (RDA) to respond significantly to the contamination (Figure 1). These groups have not been identified previously to respond to contamination; however, groups such as MND1 have been found in other contaminated environments [52]. Interestingly, many of these groups corresponded more to the presence of arsenic than chromium.

**Table 2:** Pyrosequencing results, diversity estimates, and 16S rRNA gene copy abundance for each sampling site.

		Pyrosequencing Results		Diversity Estimates^1^			16S rRNA Abundance
Site	Contamination Status	Total Sequences	Total OTUs^2^	ACE	Chao	Shannon	Log copies g^−1^dwt soil
Kala Shah Kaku	Control	15610±2730	6095±544	26008±1817	15518±806	7.9±0.01	7.45±0.05
	Cr (VI) +Ascontaminated	20907±0	3191±0	9597±0	6794±0	5.9±0	8.89±0.13
Kasur	Control	21745±4737	6555±1076	23970±3825	14908±2425	7.5±0.12	6.33±0.07
	Cr (VI) contaminated	16221±1576	3754±354	15661±868	9520±908	6.4±0.11	8.81±0.03
Sialkot	Control	28102±3860	8057±547	28055±706	17971±749	7.7±0.01	7.59±0.02
	As contaminated	23979±315	5758±567	20483±3472	13096±2024	7.3±0.11	7.98±0.03

1 Diversity Estimators Abbreviations: Abundance-based Coverage Estimator (ACE), Chao’s species richness estimator, and Shannon-Weiner Index.

2 Species level, 97% similarity threshold used to define Operational Taxonomic Units (OTUs).

Quantification of total bacterial 16S rRNA gene copies using qPCR showed that the contaminated sites in general had higher 16S rRNA gene copy abundance than did their paired controls (Table 2). Soils with Cr (VI) (Kasur and Kala Shah Kaku) had the highest 16S rRNA gene copy abundance and showed the greatest disparity in 16S rRNA gene copy number compared to their paired control (∼1.5–2 log fold differences) (Table 2).

### Shifts in Diversity Due to Contamination

**Figure 3:**
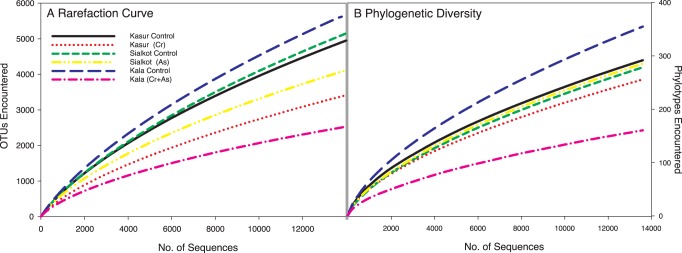
Alpha-diversity analysis using rarefaction (A) and phylogenetic diversity (B) of contaminated and control soils. Each soil is represented by color and pattern. Rarefaction analysis focuses on discovery of novel species level (97%) Operational Taxonomic Units (OTUs) while phylogenetic diversity focuses on the discovery of novel phylogenetic branches.

The contaminated sites all showed a 14 to 38% reduction in the number of total OTUs_0.03_ relative to the paired controls when corrected for the total number of sequences in each library (Table 2). The presence of both As and Cr (VI) together had the greatest effect on diversity, whereby a 38% reduction in alpha diversity was observed at the Kala Shah Kaku contaminated site. In contrast, the diversity in the Sialkot contaminated soil, which had arsenic only and in the Kasur contaminated soil, which had Cr (VI) only, declined by 14% and 24%, respectively (Table 2). Calculation of species richness (Chao), evenness (ACE), and diversity (Shannon) indices all confirmed the decrease in diversity within the contaminated soils when compared to control sites (Table 2). Species richness estimators are known to be sensitive to sample size [51], [53] however, rarefaction curves corroborated the results observed with species richness estimators. Thus exposure to arsenic, Cr (VI), and the combination of the two likely caused a marked reduction in microbial diversity as is clearly shown in rarefaction curves (Figure 3a). The decline in microbial diversity was not solely due to unequal sampling as Faith’s phylogenetic diversity (PD) is less sensitive to sampling size [54]. Phylogenetic diversity in addition to being less sensitive to sampling effort also incorporates phylogeny of OTUs such that closely related OTUs are considered redundant. Thus, a community with a larger PD will have a more phylogenetically dispersed community [54]. As and Cr (VI) had the greatest effect on PD, decreasing it by 55% (Figure 3b). The second largest decrease in Faith’s phylogenetic diversity occurred in Kasur soils, which were contaminated primarily with Cr (VI). In the arsenic-contaminated soil (Sialkot) only a 12% reduction in Faith’s phylogenetic diversity was observed, while total diversity was not (Figure 3b).

To test which environmental factors were contributing to the variation in microbial diversity, Pearson correlation coefficients were calculated. Total chromium, Cr (VI), and pH, were all significantly-negatively correlated with Chao, ACE, and Shannon metrics (P<0.05, N = 9) (Table 3, correlation coefficients are in Table S2). Total chromium was more significantly correlated with diversity than Cr (VI); however, the strongest correlation to diversity of all environmental factors tested was pH. The presence of arsenic was negatively correlated to all diversity metrics, but these correlations were not significant (Table 3). Phylogenetic diversity, despite having a negative slope with metal presence, was not significantly correlated to the type of metal contamination. The only positively correlated environmental factor was phosphates, but the correlation was not significant.

**Table 3:** Effect of metal contamination, pH and soil organic matter on diversity and evenness metrics of total soil communities^1^.

α-Diversity Metric^2^	pH	Organic Matter	As	Cr (total)	Cr (IV)
Chao	**	ns	ns	*	ns
ACE	**	ns	ns	*	*
Shannon	***	ns	ns	*	*
Phylo Diversity	ns	ns	ns	ns	ns

1 Significance was assessed using a two-tailed t-test (n = 9) and p-values are represented by: * ≤0.05, ** ≤0.01, and *** ≤0.001.

2 Abbreviations: Abundance-based Coverage Estimator (ACE), Chao’s species richness estimator, Shannon-Weiner Index, Faith’s Phylogenetic Diversity, and ns, not significantly different.

**Figure 4:**
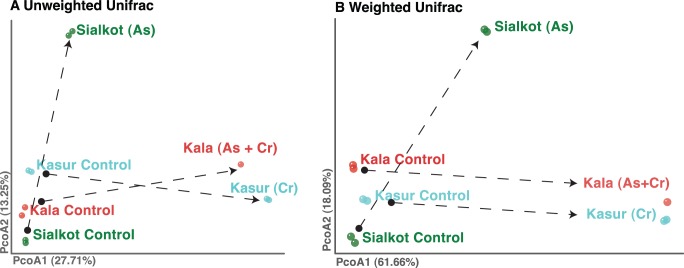
Effect of As and Cr(VI) contamination on β-diversity of the microbial communities from control and contaminated sites. The first two coordinate axes from a principal coordinate analysis (PCoA) were plotted using the unweighted (A) and weighted (B) Unifrac algorithms. Colors represent paired control and contaminated sites.

In order to compare the structure of microbial communities between sites and observe the effect of Cr (VI) and As had on community structure, beta diversity metrics were calculated. We chose to use the phylogenetic tree-based method, Unifrac, as it has been shown to be a robust measure of community similarity [48]. Unweighted (total diversity) and weighted (lineage specific diversity) Unifrac analyses suggests that chronic exposure to Cr (VI) and As has significantly altered the microbial community structure (P = 0.002 for both weighted and unweighted algorithms using an individual sample analysis with 500 permutations) (Figure 4 a & b). Visualization of weighted and unweighted Unifrac distances with PCoA analyses shows that spatial isolation has little effect on community structure especially in control plots (Figure 4); however, we add a caveat that shared species are not driving these similarities but rather the similarity at the family and genus level is. Furthermore, the community structure of the Sialkot contaminated site, which had As and not Cr (VI), was very different from the other two sites, both of which had Cr (VI) but only one of which had As (Figure 4). The presence of Cr (VI) appeared to be a greater selective force on structuring the microbial community than was As as evidenced by the clustering of Kala (Cr+As) with Kasur (Cr) contaminated sites by Unifrac analyses. Cr (VI) contamination accounted for 28 and 62% of total variance by unweighted and weighted Unifrac analysis, respectively, compared to As contamination, which accounted for 13 and 18% of total variance by unweighted and weighted Unifrac analysis, respectively.

### Comparison of *ACR3* and *arsB* Genes from Clone Libraries

**Figure 5:**
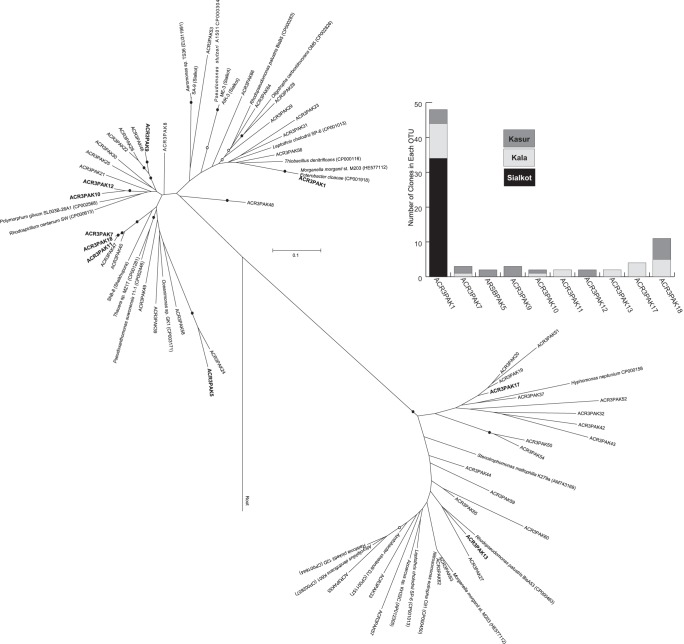
Phylogeny of *ACR*3 OTUs recovered from contaminated soil clone libraries. Neighbor-joining tree showing relationships between the *ACR3* clone library sequences, ACR3 genes from the As-resistant isolates, and closely related sequences from the NCBI database. *ACR3* sequences generated in this study are in bold and the inlaid graph shows the distribution of OTUs with more than 1 sequence.

A total of 52 different *ACR3* OTUs (Operational Taxonomic Units) were recovered from the sequencing of 125 clones. Of these OTUs, 12 were detected in Sialkot soil (As) (45 sequences), 22 detected in Kala Shah Kaku soil (As and Cr) (44 sequences) and 24 detected in Kasur soil (Cr) (36 sequences). The *ACR3* gene clone libraries from each of the three contaminated sites revealed a single *ACR3*-like OTU (Figure 5, ACR3PAK1), which accounted for over 73% of the clones from the Sialkot site, ∼22% of the clones from the Kala Shah Kaku site, and ∼10% from Kasur. Blastn and subsequent phylogenetic inference revealed that the OTU ACR3PAK1 is nearly identical to the *ACR3* gene found in the two *Gammaproteobacteria Morganella morannii* and *Enterobacter cloacae.* Two other OTUs, ACR3PAK23 and ACR3PAK56 also clustered near the *ACR3* from *T*. *denitrificans* (Figure 5). ACR3PAK18 was the second most abundant OTU, but was only detected in Kasur (Cr) and Kala Shah Kaku (As and Cr) contaminated soils. ACR3PAK18 is related to the *ACR3* gene from the marine *Alphaproteobacterium, Oceanimonas doudoroffii* forms a unique clade with other Kala and Kasur OTUs but is only 77 and 76% similar to an *ACR*3 from *Thauera* sp. MZ1T and *Rhodospirillum centenum* SW, respectively.

In contaminated soils, the diversity of *ACR3-*like genes was much higher than expected, and likely under sampled. Interestingly, few *ACR3-*like OTUs were shared across our three study sites but those that were shared were dominant at all three sites. Much like 16S libraries, many of the *ACR3-*like OTUs were categorized as rare (*i.e.* OTUs containing one to two representative sequences), and represent the bulk of the library. Furthermore many of these OTUs form unique clades including ACR3PAK17, which is loosely associated with *Hyphomonas neptunium.* Even more intriguing, these sequences were divergent from other *ACR3* genes from isolated organisms, suggesting that these genes may represent novel uncultivated organisms.

**Figure 6:**
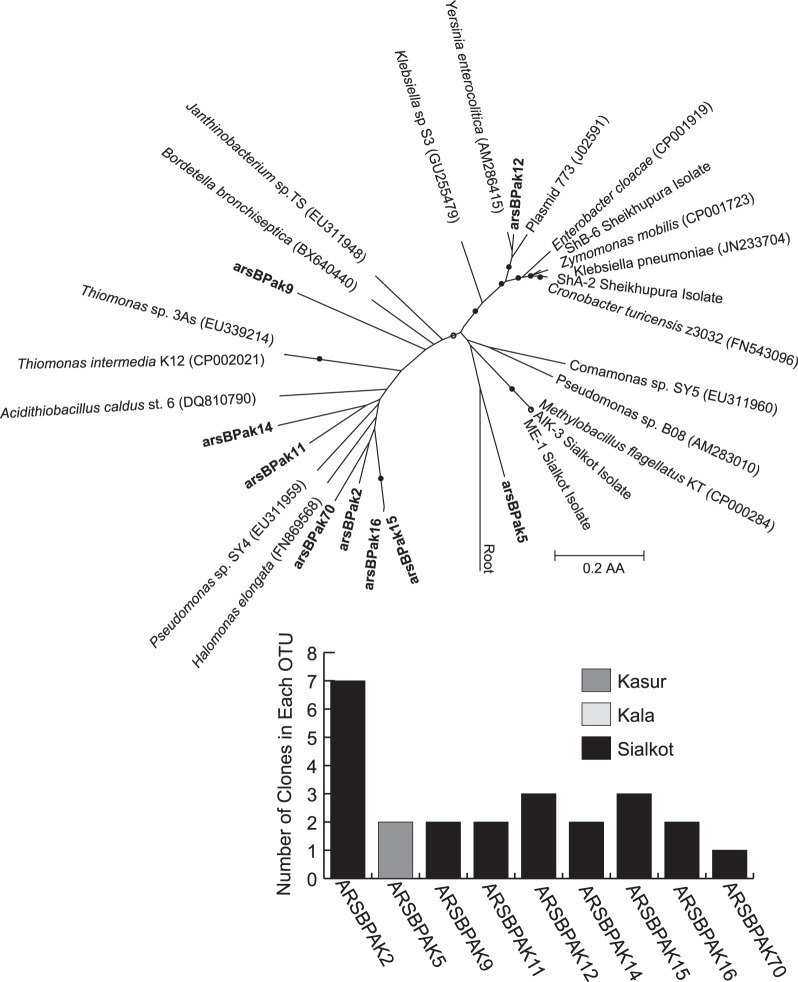
Phylogeny of *arsB* OTUs recovered from contaminated clone libraries. Neighbor-joining tree showing relationships between the *arsB* clone library sequences, *arsB* genes from As-resistant isolates, and closely related sequences from the NCBI database. *arsB* sequences generated in this study are in bold and the inlaid graph shows the distribution of OTUs with more than 1 sequence.

We sequenced 53 clones from the *arsB* gene clone library. A number of the clones were shown to have little homology to *arsB* based on Blast analysis. Among the clones shown to be *arsB* homologs, a total of 9 OTUs were recovered and the library differed from the *ACR3* library in that it contained several dominant *arsB-*like OTUs that were unique to each individual site (Figure 6). Of these OTUs, 8 were detected in the Sialkot (As) site (22 sequences), and only one was detected in the Kasur site (2 sequences). No *arsB* OTUs were recovered from Kala Shah Kaku soil. In addition, no OTUs were shared amongst all three sites. In the Sialkot (As) *arsB* library a single OTU, arsBPAK2, dominated the library and represented 32% of the total Sialkot clones. arsBPAK2 is loosely affiliated within a clade of other *arsB* OTUs which include the gene from halophilic *Halomonas elongata.* Interestingly, *Halomonas*-like sequences were also detected in pyrosequencing libraries.

### *ACR3* and *arsB* Sequences from Isolates

In order to isolate As resistant strains from contaminated soils, 1 gram of contaminated soil from study sites near Kala (Sheikhupura) and Sialkot was serially diluted in 0.1% sterile saline and plated onto nutrient agar supplemented with 5 mM sodium arsenate. Diverse colony morphologies were chosen for further isolation and screened for arsenic resistance on Davis minimal broth [55] supplemented with 5 mM arsenic. A total of seven As resistant isolates (Table 4) were cultivated from contaminated soils near sites assessed by pyrosequencing. These were then screened for the presence of *ACR3* and *arsB* genes (Table 4). All isolates obtained belong to the *Alphaproteobacteria* and *Gammaproteobacteria*. Only one isolate (AIK-3) contained both the *ACR3* and *arsB* genes. None of the *ACR3* or *arsB* genes from isolates were similar to any of the OTUs obtained from clone libraries. Phylogeny of each isolate’s 16S rRNA gene and *ACR*3 or *arsB* genes did not agree, which reaffirms that arsenic resistance genes are highly mobile with the microbial community.

**Table 4:** Presence of *arsB* and *ACR3* in arsenic resistant isolates from Pakistani soils.

Isolate ID	Most Similar Type Strain	Similarity (%)^1^	Site	*arsB*	*ACR3*
**SHB-6**	*Enterobacter cloacae* str. ATCC 13047T	99.4	Sheikhupura	+	−
**SHB-8**	*Rhizobium selenireducens* str. B1	98.5	Sheikhupura	−	+
**SA-9**	*Aeromonas punctata* str. NCIMB 13016	99.8	Sialkot	−	+
**SH-A2**	*Klebsiella pneumonia* str. ATCC 13884T	98.3	Sheikhupura	+	−
**AIK-3**	*Pseudomonas stutzeri* str. ATCC 17588	100	Sialkot	+	+
**ME-1**	*Pseudomonas stutzeri* str. ATCC 17588	100	Sialkot	+	−
**ME-3**	*Rhodococcus pyridinivorans* str. PDB9	99.8	Sialkot	−	+

1 Similarity assessed using RDP Seqmatch function on type strain sequences.

### T-RFLP Analysis and Diversity Analysis

**Figure 7:**
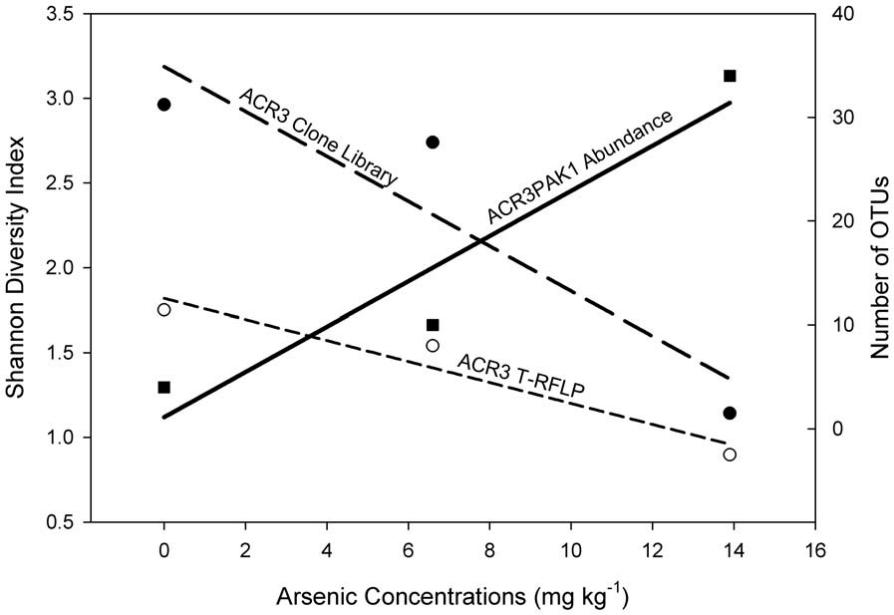
Diversity of *ACR3* genes versus increasing arsenic concentrations. Graph shows the Shannon Weiner diversity indices for the clone libraries and T-RFLP results of the *ACR3* gene, as well as the arsenic concentration of the three sample sites. The figure illustrates the diversity trends observed for *ACR3* across the three sites, and how they relate to the most dominant OTU in the library. Shown are the diversity values for all three sites from Clone Library data, from T-RFLP data, and the abundance values for the most dominant OTU, ACR3PAK1.

As was observed in the *ACR3* clone analysis, T-RFLP results showed the *ACR3* gene was quite diverse when all sites were considered. However patterns of diversity were distinct for each site and inversely correlated with increasing arsenic concentrations (Figure 7). Diversity of *arsB* through T-RFLP was not included in this study due to the apparent non-specific amplification from the *arsB* primer set. Thus, the diversity of *arsB* genes was determined only from clone libraries and shows a positive increase in diversity as arsenic concentrations increase (Figure 6). However, due to the low recovery of *arsB* sequences from clone libraries, we add the caveat that further sequencing or development of more specific primers are needed to fully elucidate the *arsB* diversity pattern.

## Discussion

In Pakistan, the extensive use of chromium and arsenic by the leather tanning industry and historical lack of environmental regulation has left soils near metropolitan areas inundated with these hazardous metal contaminants. Bioremediation strategies targeting metal transforming bacteria are often chosen due to their relatively low cost and environmental impact when compared to chemical treatments [56]. However, stimulating microbially mediated metal reduction will have competing outcomes. Although the reduction of Cr (VI) to Cr (III) leads to detoxification and precipitation, As (III) is more mobile and toxic than As (V). Thus, it is important to understand the effects of long term arsenic and chromium contamination on the resident microbial communities to develop effective remediation strategies.

Alpha diversity (*i.e.* the species richness and evenness within a sample) of microbial communities has often been correlated with ecosystem stability and functionality [57], [58]. In our study, we observed a marked reduction in bacterial alpha diversity (Shannon Index) in each of our contaminated soils relative to their paired control (Table 2). Reduction in alpha diversity has been shown in metal contaminated sediments for *Archaea* [34] and in soils for *Bacteria* [1]. Odum [59] suggested that the reduction in diversity is rooted in the organisms’ inability to physiologically cope with the stressor and, in these soils, the presence of both Cr (VI) and arsenic had the most pronounced effect on diversity (Figure 2). Furthermore, soils where Cr (VI) was present (Kala Shah Kaku and Kasur) saw a marked reduction in phylogenetic diversity especially when compared to the soil containing only As (Sialkot) (Figure 3). The reduction in alpha diversity was likely not pH dependent as pH differences were relatively small between As and Cr contaminated soils. Significant correlations with pH and alpha diversity were not due to shifts in pH but rather the discernable difference between controls and contaminated soils (Table 3). Furthermore, since both control and contaminated soils had circa-neutral pH, large differences in alpha diversity would not be anticipated as neutral pH soils have been shown to be relatively similar in total diversity [33]. Thus, the reduction in total diversity and phylogenetic diversity in the presence of either contaminant suggests that many microbial species could not cope with the long-term Cr (VI) or As stress. This may be because the resistance genes for chromium (VI) are less mobile throughout the community than arsenic resistance genes [30] or that chromium at the ambient concentrations exerts a higher level of stress on individual community members.

Phylogenetic analysis of species level OTUs (97% similarity cutoff for Operational Taxonomic Units) revealed a significant shift in dominance from *Actinobacteria* in control soils to *Proteobacteria* in contaminated soils (Figure 3). The shift in phylum level dominance was independent of the site and suggests that *Proteobacteria,* as a group, may be the most metal tolerant organisms found at metal contaminated sites. Additionally, all except one (*Rhodococcus*) of the isolated As resistant species were determined to be *Proteobacteria*. Previous chromium and arsenic studies showed that *Proteobacteria* capable of metal transformation are routinely cultivated [8] and identified using molecular tools [60]–[62]. Additionally, Odum [59] suggests that r-selected organisms (rapidly reproducing), such as *Proteobacteria* [63], [64], are favored after a stressor is applied to an ecosystem, which is perhaps a reason for their dominance. The distribution patterns of *Proteobacteria* differed between sites with either *Alphaproteobacteria* or *Gammaproteobacteria* being dominant (Figure 2) suggesting that these groups differentially respond to either chromium or arsenic. Several novel proteobacterial groups specifically LO11, MND1 and WJ2 were predominant in contaminated soils and heavily influenced by the presence of arsenic. Furthermore the occurrence of novel arsenic resistance gene clades (*ACR*3 and *ars*B) from contaminated sites implies the rare biosphere may harbor phylogenetically distinct resistance mechanisms. The presence of these groups suggests that novel microorganisms may play an important role in contaminated soils and that their presence may also provide a stabilizing element as these novel but abundant groups can be highly adapted to dealing with extreme environments [52].

It is unclear whether the decrease of *Actinobacteria* is attributed to chromium or arsenic toxicity. Some *Actinobacteria* have been implicated in metal cycling [65], but the *Actinobacteria* could have been negatively impacted by higher than normal soil moisture [66], as the contaminated soils were routinely exposed to waste effluents. The loss of *Acidobacteria* in contaminated soils may stem from an increased pH rather than the presence of Cr (VI) as pH is known to be a strong regulator of *Acidobacteria* abundance and diversity [67]. The loss of diversity among k-selected groups (highly adapted ecological groups) such as *Actinobacteria* and *Acidobacteria* [64] may ultimately decrease the stability of the system, particularly because these groups are thought to produce extracellular enzymes necessary for complex carbon degradation that likely supports growth of other microorganisms [68], [69]. Nevertheless, the prominent shift in phylogeny towards *Proteobacteria* suggests that resistance to chromium and arsenic are widespread within this phylum, and that novel families within the *Proteobacteria* may play important ecological roles.

It is clear that in these soils, chronic exposure to chromium and arsenic reduced diversity and shifted phylogeny (Figures 2 & 3 respectively). However, we were also interested in whether the structure of bacterial communities is conserved over a wide geographic area, especially in response to exposure to contaminants. Past work showed that few species are shared across spatially separated soils [24]. Yet environmental factors, such as pH [33] and salinity [70], have been shown to exert selective pressures on the microbial community, which increase the similarity between spatially isolated communities. In this study, the beta-diversity (*i.e.* community similarity between sites) of control soil communities was remarkably similar to each other despite being spatially separated. The bacterial communities in soils with Cr (VI) were more similar to each other than to the Sialkot bacterial community, whose soil contained As but not Cr (VI) (Figure 4). The similarity of the bacterial communities in Cr (VI) contaminated soils, despite their spatial isolation, suggests that chromium resistance may be acquired, as is the case with arsenic resistance. However, due to the highly oxidizing nature of Cr (VI), resistance may arise through non-specific interactions with proteins or secondary metabolites capable of donating electrons to Cr (VI). Thus, similar communities could arise if the genes responsible for the formation of the proteins/metabolites are phylogenetically conserved at the genus and potentially family level, which is also a principal driver of beta diversity similarity. Furthermore, because chromium is essentially inert after reduction from oxidation state VI to III, specific and conserved efflux pumps that are commonly associated with arsenic resistance would not be required for chromium resistance.

The *ACR3* gene was shown to have more singularly dominant OTUs (Figure 5) and less diversity (Figure 7) when exposed to higher arsenic concentrations. The drop in diversity of *ACR3* genes at higher arsenic concentrations suggests that only a fraction of *ACR3* proteins are able to function at the higher arsenic concentrations. A study done with arsenic contaminated soils in China [71] showed that isolated bacteria containing *ACR3* genes demonstrated a higher tolerance for arsenic compared to those containing only *arsB*. This suggests that high arsenic environments would favor *ACR3*, however, the high arsenic levels observed appeared to favor only a specific variant of *ACR3*, the ACR3PAK1 OTU. It is unclear from our data whether this dominance of a single *ACR3* variant is due to an abundance of a single organism or if many organisms have acquired this variant from a horizontal gene exchange. Due to the previously mentioned limitations of the *arsB* primers, similar diversity analyses with clone libraries and T-RFLP could not be accurately obtained. However, an observed positive correlation between increasing arsenic concentrations and *arsB* diversity was observed. The differential response of *ACR3* and *arsB* diversity under increasing arsenic concentration is puzzling as both genes are generally found on the same operon and warrants further study.

This study sought to understand the spatial relationships of bacterial communities exposed to chromium and arsenic and the effect that long-term metal exposure has on the community structure, diversity and cell abundance. Despite being spatially isolated, soils exposed to chromium (VI) were similar in structure and each saw a large reduction in diversity. Interestingly, Cr (VI) presence appears to be a much stronger selector of community structure than As. Nonetheless, the marked reduction in microbial diversity across all contaminated soils and changes in phylogeny observed in the soils will likely have important effects on the ecosystem function and ultimately the restoration of the biome, as the physiological diversity of these soil microbial communities was also likely affected. However, inferring the functionality of the community is not possible at this point, as many of the bacteria identified in our study are uncultivated. The physiological response of these microbial communities as remediation and ultimately restoration of the biome proceeds will depend on the intrinsic genetic potential that still remains within the microbiome.

## Supporting Information

Table S1 Geochemical characterization of study site.
(DOCX)

Table S2 Correlation coefficients, T-statistics, and significance values of alpha diversity metrics across the contamination gradient.
(DOCX)
