# Supplementary material for: Exposure of Soil Microbial Communities to Chromium and Arsenic Alters Their Diversity and Structure
Source: PLoS One. 2012 Jun 29;7(6):e40059. doi: 10.1371/journal.pone.0040059 (PMC3386950; doi:10.1371/journal.pone.0040059)
Supplement: Table S1 — Geochemical characterization of study site. (DOCX) [file pone.0040059.s001.docx]

|  | **Kala Shah Kaku** | | **Kasur** | | **Sialkot** | |
| --- | --- | --- | --- | --- | --- | --- |
|  | Control | Cr+As | Control | Cr-Only | Control | As-Only |
| pH | 6.5 | 7.4 | 6.4 | 8 | 6.8 | 7 |
| EC (ms•cm^-1^) | 28.7 | 12.5 | 40.2 | 15.1 | 37.5 | 9.9 |
| ≥2mm (%) | 41.3 | 33.3 | 39.6 | 23.1 | 31.4 | 43.6 |
| 1-2mm (%) | 19.8 | 14.6 | 20.4 | 13.7 | 20.8 | 15.5 |
| 500µm-1mm (%) | 13.8 | 12.9 | 12.6 | 11.1 | 15.3 | 14.8 |
| 250-500µm (%) | 12.1 | 12.2 | 13.2 | 9.4 | 15.0 | 15.0 |
| 125-250µm (%) | 8.9 | 8.9 | 9.7 | 9.0 | 10.2 | 8.8 |
| 63-125µm (%) | 2.2 | 4.6 | 2.8 | 7.9 | 3.3 | 1.2 |
| 0-63µm (%) | 1.2 | 13.4 | 1.6 | 25.4 | 3.5 | 1.1 |
| Sodium (g•kg^-1^) | 5.2 | 1.1 | 5.4 | 1.4 | 4.6 | 0.6 |
| Potassium (g•kg^-1^) | 0.1 | 0.05 | 0.1 | 0.09 | 0.2 | 0.03 |
| Calcium (g•kg^-1^) | 2.3 | 0.9 | 2.2 | 1.0 | 1.9 | 1.1 |
| Magnesium (g•kg^-1^) | 0.6 | 0.3 | 0.6 | 0.4 | 0.7 | 0.9 |
| Sulfates g•kg^-1^) | 1.9 | 2.3 | 2.8 | 3.2 | 2.2 | 2.1 |
| Carbonates (g•kg^-1^) | 0 | 0 | 0 | 0 | 0 | 0 |
| Bicarbonates (g•kg^-1^) | 1.8 | 0.3 | 1.2 | 0.61 | 0.96 | 0.5 |
| Organic matter (%) | 5.4 | 4.5 | 3.6 | 7.9 | 4.9 | 3.4 |
| Phosphates (mg•kg^-1^) | 35.6 | 18.6 | 46 | 20.3 | 31.6 | 0 |
| As (mg•kg^-1^) | 0 | 6.6 | 0 | 0 | 0 | 13.9 |
| Cr (VI) (mg•kg^-1^) | 0 | 1.2 | 0 | 4.2 | 0 | 0 |
| Total Cr (g•kg^-1^) | 3.82 | 6.24 | 1.61 | 24.8 | 2.46 | 0.23 |
