# Supplementary material for: Exposure of Soil Microbial Communities to Chromium and Arsenic Alters Their Diversity and Structure
Source: PLoS One. 2012 Jun 29;7(6):e40059. doi: 10.1371/journal.pone.0040059 (PMC3386950; doi:10.1371/journal.pone.0040059)
Supplement: Table S2 — Correlation coefficients, T-statistics, and significance values of alpha diversity metrics across the contamination gradient. (DOCX) [file pone.0040059.s002.docx]

| **Correlation Coeffecient** | **pH** | **OM** | **As** | **Cr total** | **C(IV)** |
| --- | --- | --- | --- | --- | --- |
| Chao | -0.73 | -0.35 | -0.26 | -0.66 | -0.58 |
| ACE | -0.73 | -0.29 | -0.32 | -0.62 | -0.53 |
| Shannon | -0.84 | -0.41 | -0.21 | -0.73 | -0.64 |
| Phylo Diversity | -0.59 | -0.09 | -0.23 | -0.40 | -0.29 |
|  |  |  |  |  |  |
| **T- Statistic (n=9)** |  |  |  |  |  |
| Chao | -3.21 | -1.12 | -0.82 | -2.64 | -2.14 |
| ACE | -3.20 | -0.91 | -1.00 | -2.38 | -1.87 |
| Shannon | -4.58 | -1.34 | -0.64 | -3.20 | -2.49 |
| Phylo Diversity | -2.21 | -0.27 | -0.70 | -1.32 | -0.92 |
|  |  |  |  |  |  |
| **P-Value** |  |  |  |  |  |
| Chao | 0.01 | ns | ns | 0.05 | ns |
| ACE | 0.01 | ns | ns | 0.05 | 0.02 |
| Shannon | 0.001 | ns | ns | 0.02 | 0.05 |
| Phylo Diversity | ns | ns | ns | ns | ns |
